# Aberrant Right Subclavian Artery with a Bicarotid Trunk: The Importance of Diagnosing This Rare Incidental Anomaly

**DOI:** 10.7759/cureus.6094

**Published:** 2019-11-08

**Authors:** Nina Hanžič, Urban Čizmarević, Vesna Lesjak, Primož Caf

**Affiliations:** 1 Radiology, University Medical Centre Maribor, Maribor, SVN

**Keywords:** aberrant right subclavian artery, bicarotid trunk, interventional cardiology, interventional neuroradiology

## Abstract

We present the case of a 69-year-old woman with a history of kyphoscoliosis, arterial hypertension, and chronic hypercapnic respiratory failure. She underwent the computed tomography of the chest, and incidental anomalies of the aortic arch branches were found. Asymptomatic aberrant right subclavian artery and bicarotid trunk, which was found, are rare and usually incidental findings. The presence of this anomaly is becoming increasingly important to interventional cardiologists and radiologists as the number of endovascular procedures is increasing every year.

## Introduction

An aberrant right subclavian artery (ARSA) presents a variation of the aortic branches and arises as the last branch of the aortic arch [1]. It may coexist with a bicarotid trunk, but this is extremely rare, with an estimated prevalence of <0,05%. The ARSA is typically asymptomatic and is usually revealed incidentally by radiological investigations [2]. In most cases, it courses posterior to the trachea and esophagus, and in rare cases, causes symptoms due to compression of these structures [2,3]. Variants of the aortic arch have been more frequently identified owing to the increasing use of imaging studies [4]. More than their local compression syndromes, awareness of and familiarity with the anatomy of ARSA and bicarotid trunk seems increasingly important due to the increased use of endovascular coronary and neurovascular procedures [5]. This report presents a case of a 69-year-old woman who underwent the computed tomography (CT) of the chest.

## Case presentation

**Figure 1:**
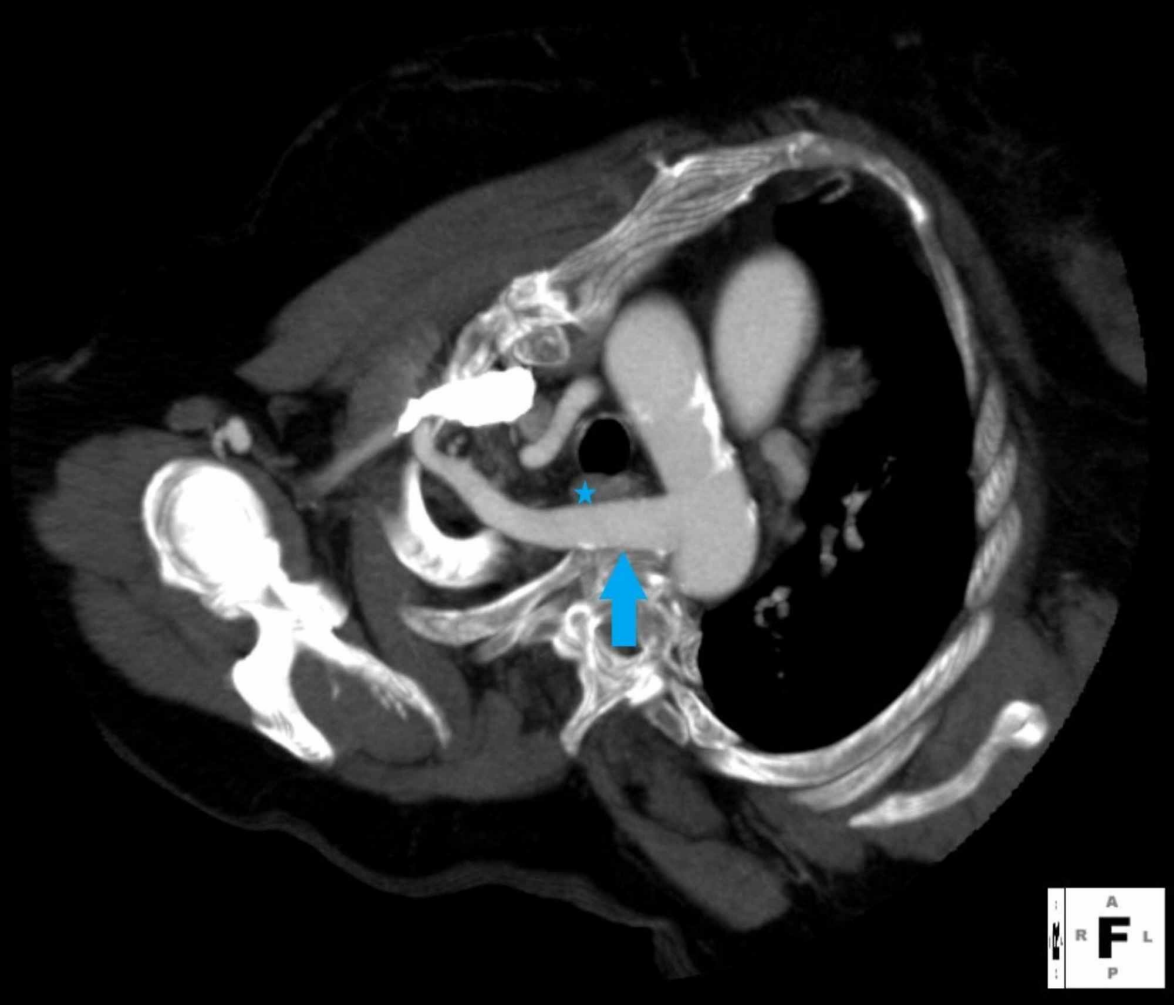
Chest contrast-enhanced reconstructed computed tomography (CT) in the venous phase, axial view, showing an aberrant right subclavian artery (arrow), which passes behind the esophagus (asterisk) and trachea.

**Figure 2:**
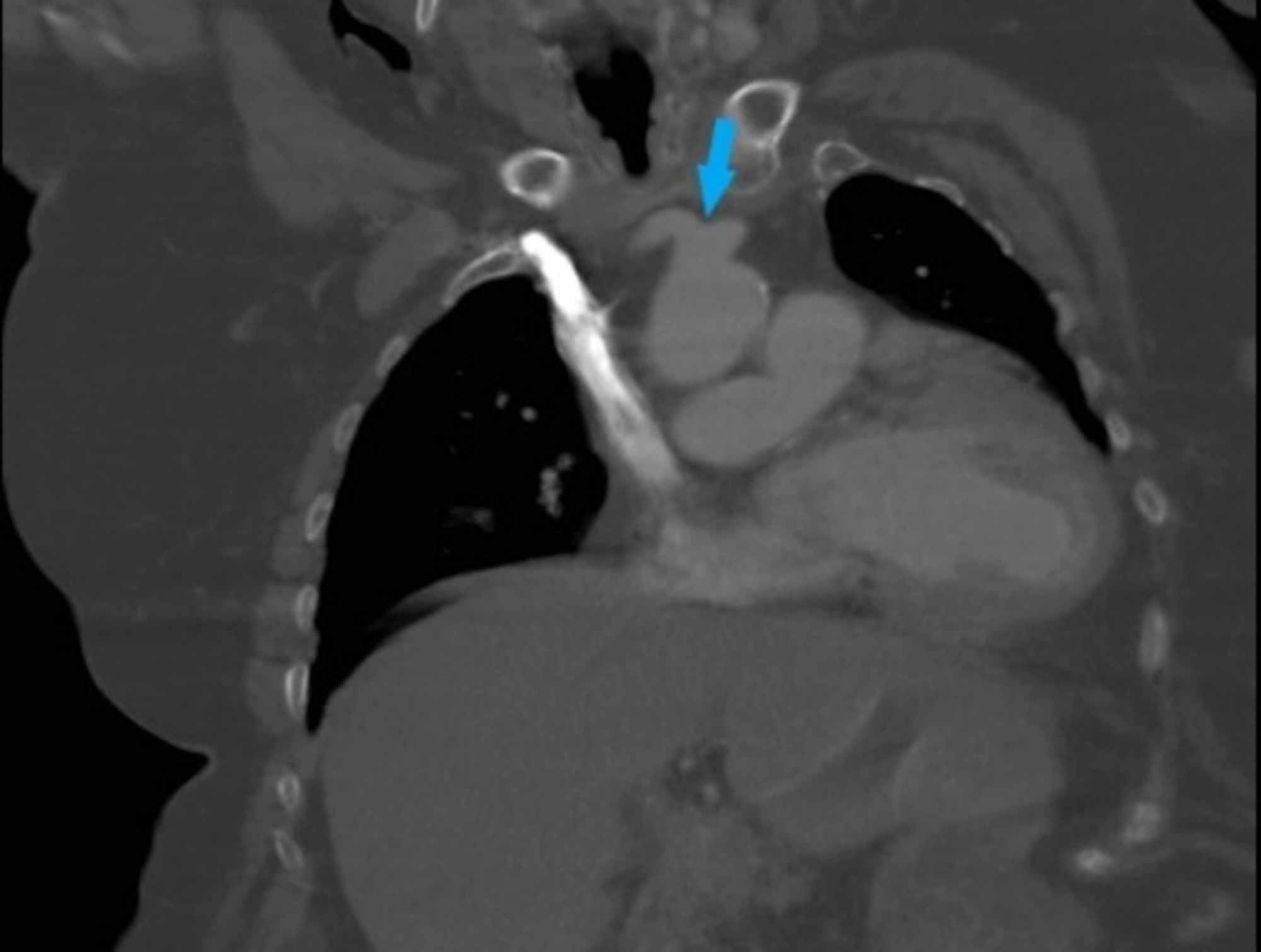
Chest computed tomography (CT), coronal view, showing a bicarotid trunk (arrow).

**Figure 3:**
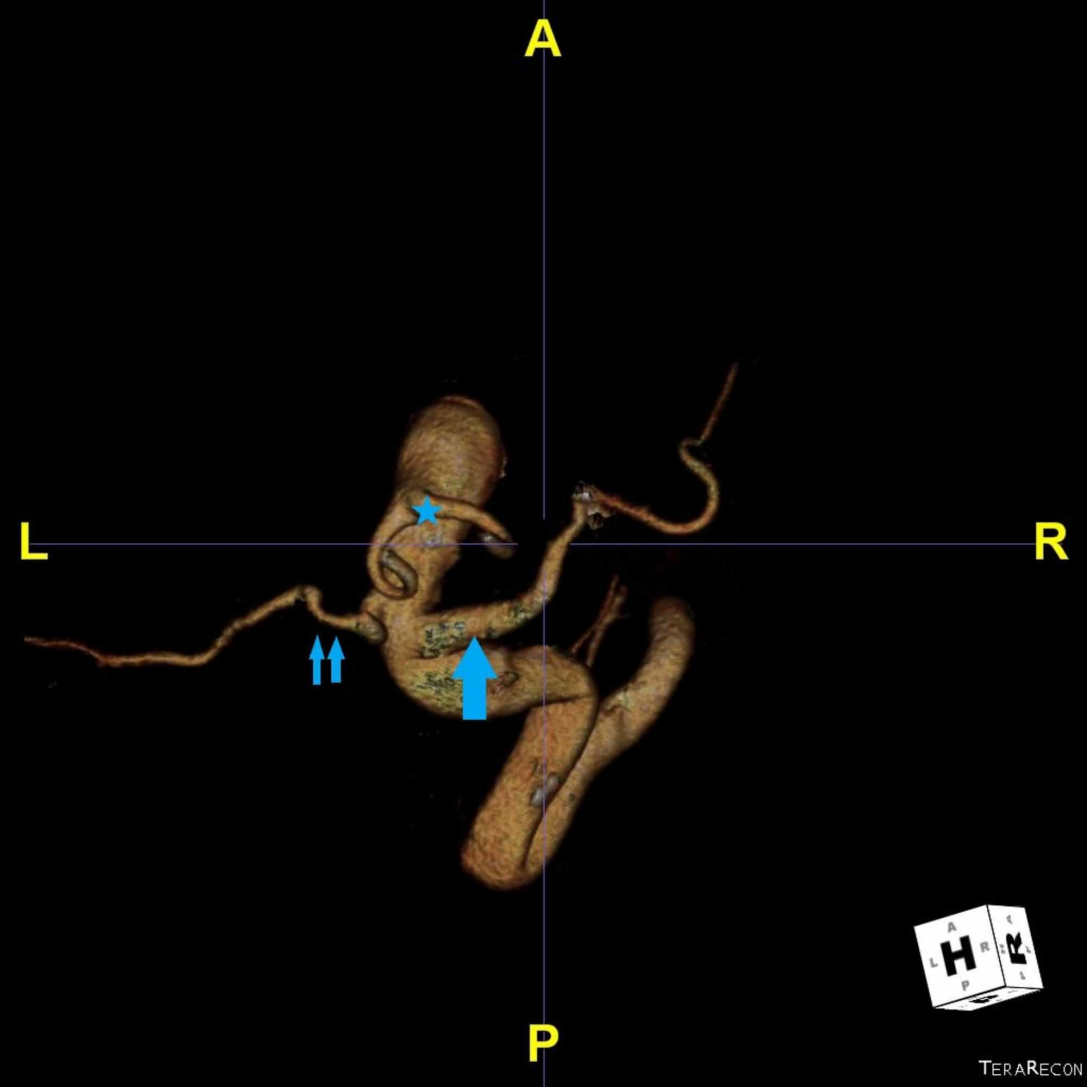
Three-dimensional (3D) reconstruction of the aorta in the cranioposterior perspective, showing a bicarotid trunk (asterisk), left subclavian artery (small arrows) and aberrant right subclavian artery (big arrow).

**Figure 4:**
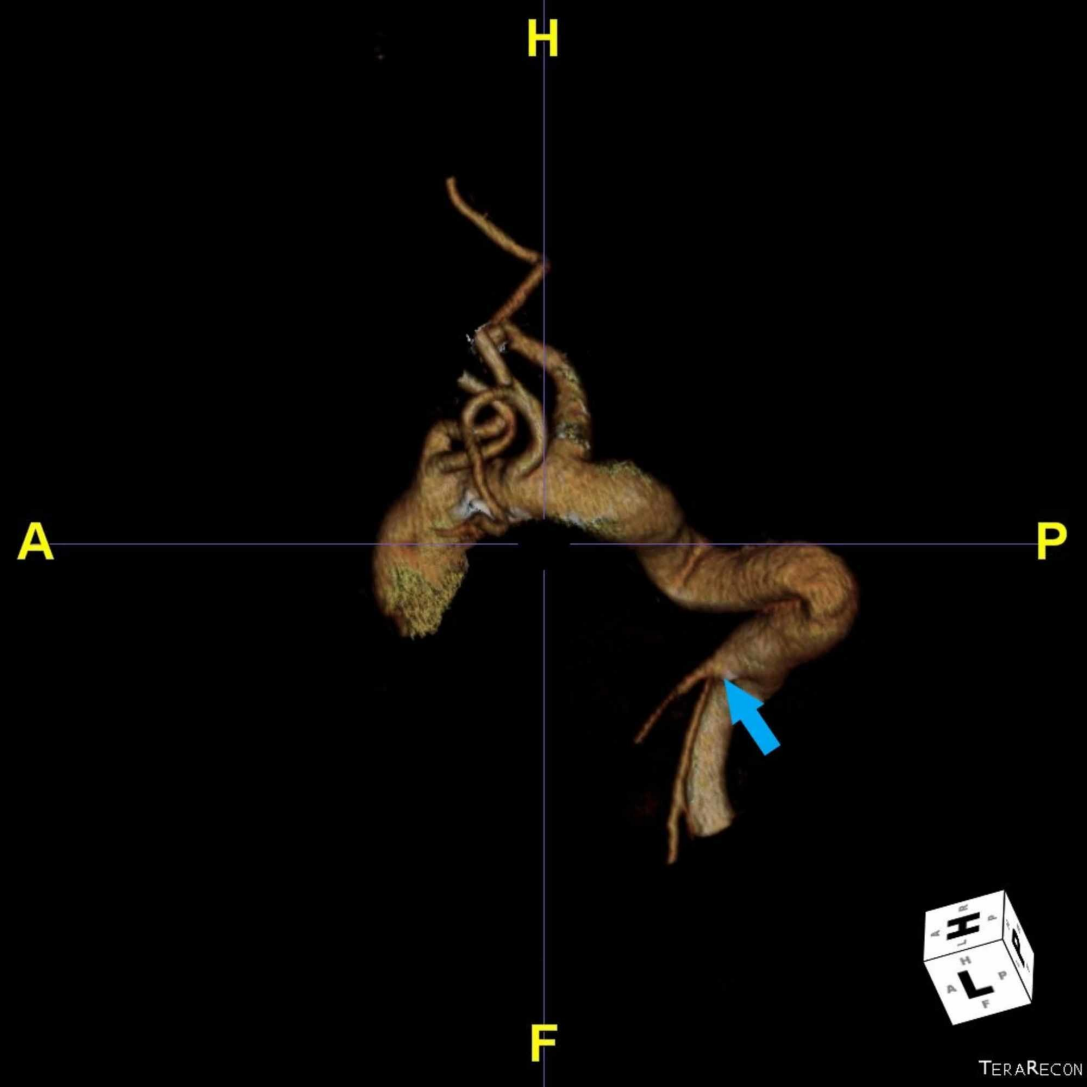
Three-dimensional (3D) reconstruction of the aorta showing branches arising from aorta, as first bicarotid trunk, as second left subclavian artery and as third aberrant right subclavian artery. The celiacomesenteric trunk (arrow) is also present.

**Figure 5:**
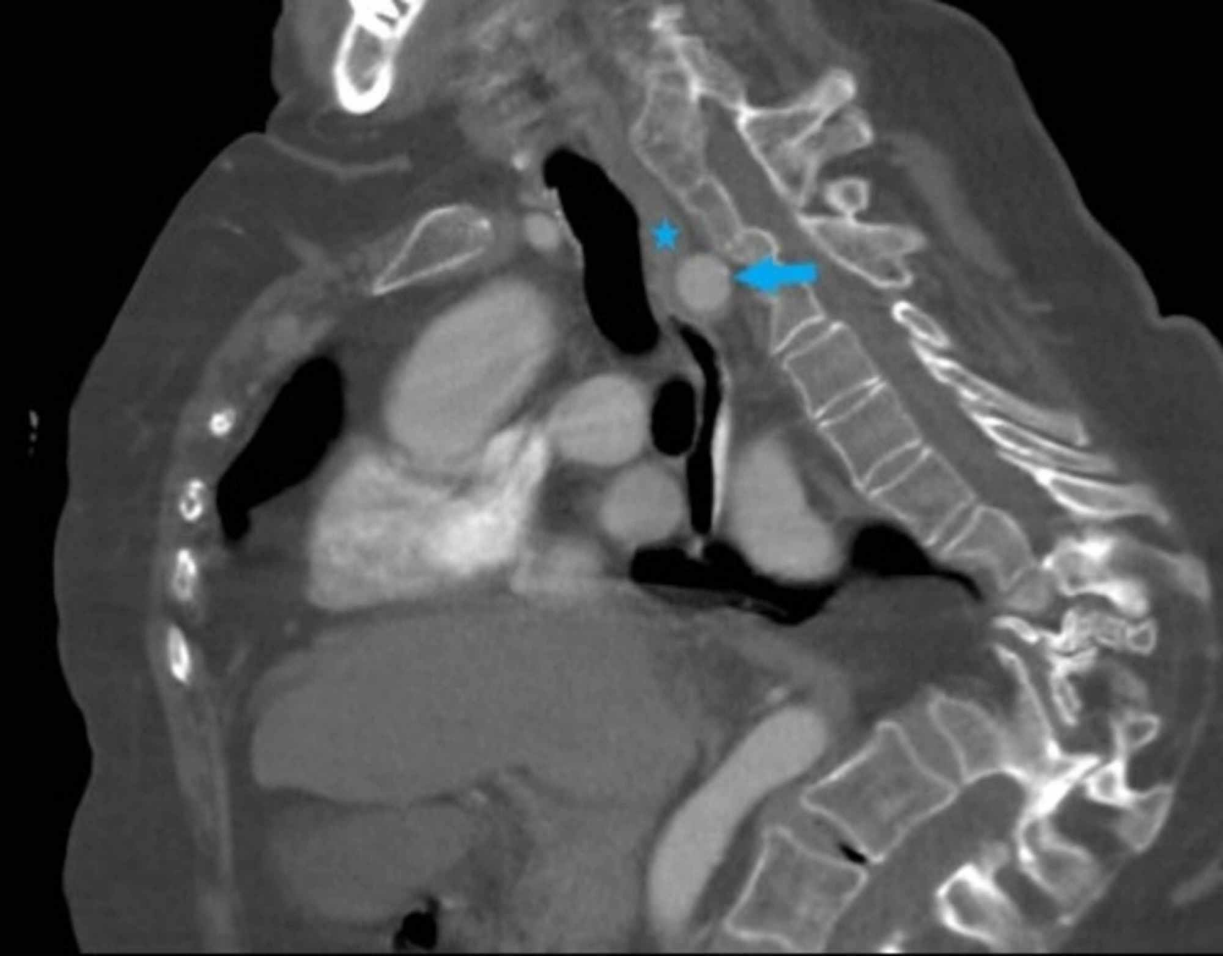
Chest contrast-enhanced computed tomography (CT) in the venous phase, sagittal view, showing an aberrant right subclavian artery passes behind the esophagus (asterisk) and trachea.

**Figure 6:**
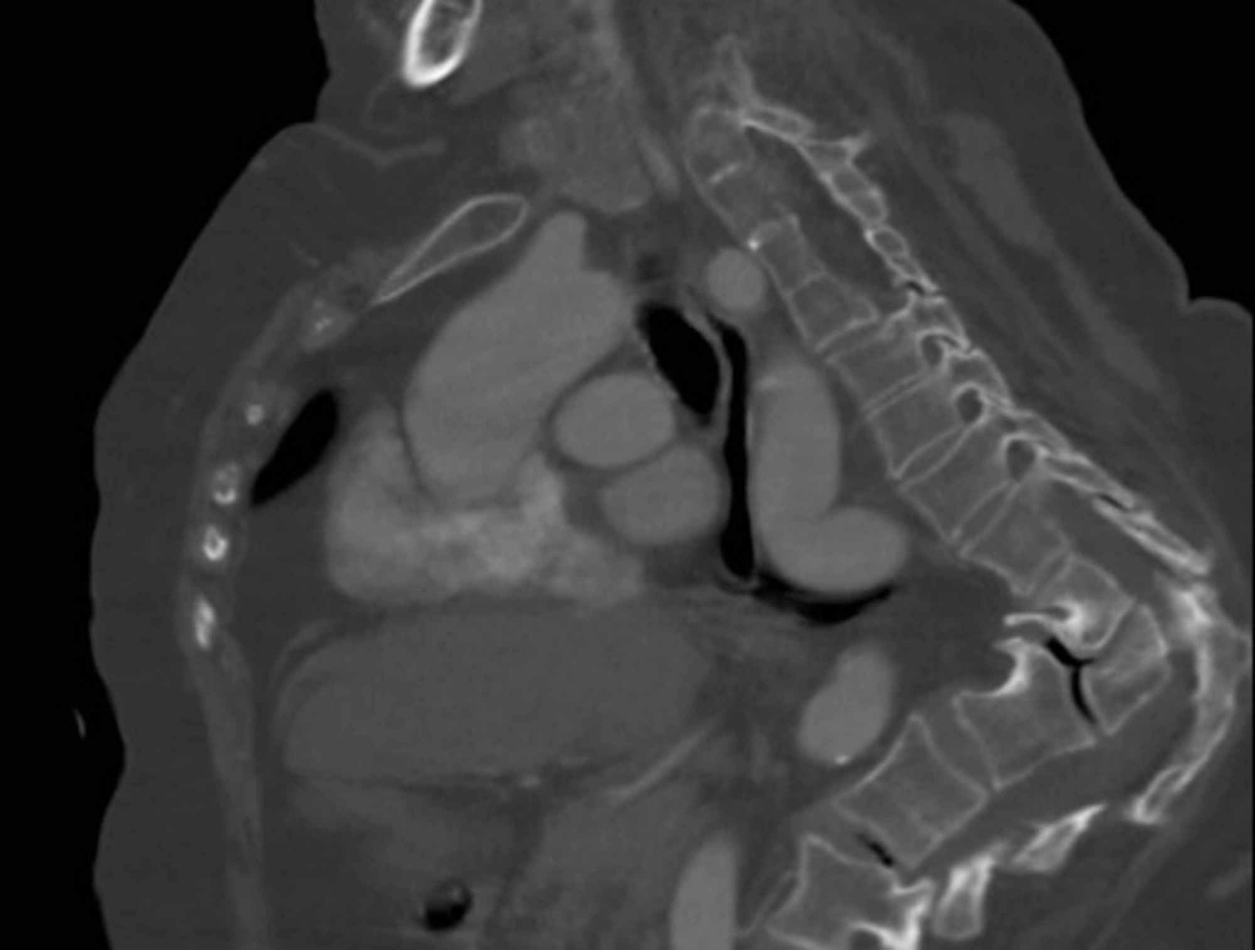
Chest computed tomography (CT), sagittal view, showing severe kyphoscoliosis of the spine.

**Figure 7:**
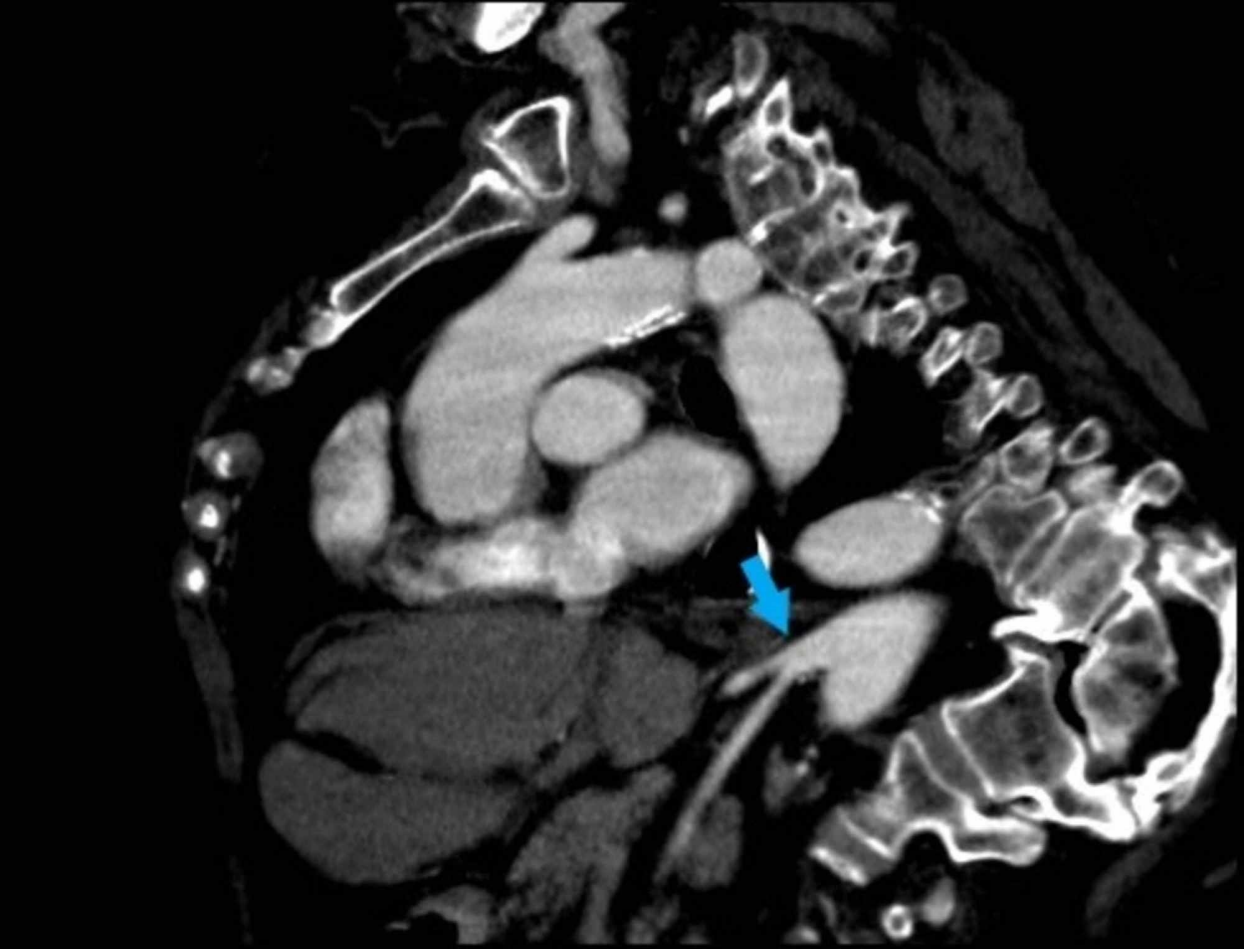
Chest and upper abdomen contrast-enchanced computed tomography (CT) in the venous phase, sagittal view, showing a common celiacomesenteric trunk (arrow).

A 69-year-old woman with a history of kyphoscoliosis, arterial hypertension, and chronic hypercapnic respiratory failure had a CT of the chest due to changes on the chest radiograph, which revealed consolidation behind the heart, and CT of the chest was suggested. The contrast-enhanced CT images in the venous phase showed anomalies of the aortic arch branches (Figures 1, 2). For better anatomical visualization, we did the Three-dimensional (3D) reconstructions of the aorta (Figures 3, 4). The first branch of the aortic arch was a bicarotid trunk, which divides into the right and left common carotid artery. The next branch was the left subclavian artery, and last was an ARSA (Figure 3). It passes posterior to the esophagus and trachea to the right upper limb (Figure 5). When analyzing the images, aneurysmal dilatation of the ARSA was not detected. Furthermore, kyphoscoliosis of the spine was observed (Figure 6). On the chest CT, which also included the upper abdomen, a cealiacomesenteric trunk as a coincidental finding was depicted (Figures 4, 7). The radiological report was saved in the hospital database and can be retrieved in the case of any interventions in the future. To our knowledge, no further check-up or evaluation by the dedicated specialist (cardiac surgeon) was done. The patient also received the radiological report and was instructed to inform the physicians of the anomaly by herself as well. Due to the lack of any symptoms associated with the anomaly, further follow-up was not performed.

## Discussion

Aorta is the main artery originating from the left ventricle and is divided into ascending and descending components [3]. In approximately 80% of individuals, three branches arise from the aorta. Brachiocephalic trunk as the first branch divides into the right subclavian artery and right common carotid artery [1,4]. The left common carotid artery is the second branch, and the left subclavian artery is the last branch [1]. Aberration of the normal embryological development of the primitive aortas and aortic arches leads to the formation of an ARSA [2]. During the sixth to eighth week of gestation, the development of the aortic arches occurs [3]. The proximal part of the right subclavian artery originates from the fourth right aortic arch artery and distal part from the involution of the right dorsal aorta and right seventh intersegmental right arteries, which originate from the descending aorta [1,6]. At the absence of the fourth right aortic arch, the seventh intersegmental artery remains affixed to the aortic arch in the descending part, and ARSA arises as a last aortic branch [1,3]. The ARSA, in most cases, passes posterior to the trachea and esophagus [3]. On the other hand, bicarotid trunk is an extremely rare anomaly of the aortic arch with a reported prevalence of <0.1% and develops because the third pair of primitive aortic arch persists [3,4].

An ARSA is typically asymptomatic [7]. In a situation where bicarotid trunk and ARSA are both present, symptoms may occur. They cause restriction to the anterior movement of the trachea and esophagus as they pass anteriorly and posteriorly in relation to these structures. Also, the symptoms may occur if an aneurysm of the origin of the aberrant vessel is present [8]. The most common symptom is dysphagia due to the retroesophageal course, also clinically termed as dysphagia lusoria [1,9]. If ARSA passes between the trachea and esophagus, dyspnea may occur [1]. In our case, the patient had a history of respiratory problems, which were more likely a consequence of her severe kyphoscoliosis. Other symptoms that could indicate the diagnosis were not present.

The best imaging methods, considered as the gold standard for the diagnosis, are CT and magnetic resonance. They confirm the diagnosis and offer a detailed visualization of the aortic arch anatomy [6,10]. In radiological practice, variations of the aortic arch and vessels are often encountered [4]. The ARSA and bicarotid trunk are often an incidental finding during the imaging for other reasons, as it was in our case [2]. We made the diagnosis with the contrast-enhanced CT of the chest, observing a bicarotid trunk arising first from the aortic arch, the second branch was the left subclavian artery, and the third and last was the ARSA, passing to the right behind the esophagus. When ARSA is diagnosed as the cause of dysphagia, the artery is clinically known as arteria lusoria [1,7]. The word originates from Latin lusoria, namely, *lusus naturæ*, which could be translated as sports of nature or natural anomaly, referring to the anomalous course of arteria lusoria and its associated dysphagia [5].

This combination of variants is especially important for interventional cardiologists and radiologists [7]. The transradial approach (TRA) as a treatment of choice for acute coronary disease is being widely used in the last years. It significantly lowers the risk of access site complications compared to the transfemoral approach and is, therefore, becoming a preferred access site [9]. The right TRA is more commonly used in the majority of interventions [11]. The TRA coronary procedures with the presence of ARSA may result in unforeseen problems; one of them is an injury of the vessel. According to the literature, when ARSA was present, and a coronary procedure was performed, a TRA was successful in only 60% of procedures [7].

The number of interventions on the carotid and intracranial vessels is also increasing. In the last years, thrombectomy turned out as a cornerstone of acute ischemic stroke management and is becoming increasingly used in patients with an occlusion of the large vessel [12]. If awareness of this aortic arch anomaly is considered, the fluoroscopic time and amount of the contrast medium used in neurointerventional procedures is reduced [13,14].

Therapy for ARSA and bicarotid trunk is usually not required. In the setting of symptoms due to esophageal or tracheal compression, surgical or endovascular interventions could be used [1]. The therapy of choice would be endovascular treatment [5].

## Conclusions

The combination of ARSA and bicarotid trunk is a rare, mostly asymptomatic finding and usually presents itself as an incidental finding during the diagnostic procedures of other conditions. The number of coronary and neurointerventional procedures is increasing. Interventional cardiologists and radiologists must be aware of a possible combination of ARSA and bicarotid trunk, as it can have a considerable impact on endovascular procedures, where the right transradial approach is more commonly used. Therefore, when vascular anomalies are depicted on different imaging modalities, they should be included in the radiological report, so that therapeutic complications and procedure prolongation in eventual coronary and neurovascular interventions can be avoided.
